# Mr. Grose, on Yeast in Typhus

**Published:** 1800-12

**Authors:** I. H. Grose

**Affiliations:** Winslow


### *To the Editors of the Medical and Phyfical Journal.*

GENTLEMEN,

HAVING been favoured with the infertion of my laft letter relative to the ufe of flos cerevifiæ in Typhus, I beg leave to fubmit the following pages to your confideration; if you think them worthy of notice, they are at your difpofal.

Accounts of cafes, when faithfully related, are nearly as fatisfactory, whether the treatment has been fuccefsful or not, as, in the one inftance, the public have a remedy before them, or otherwife a ftimulus to difcovery is excited by the failure of adopted adopted remedies. The fubject on which I fhall folicit your attention is, the ufe of yeaft, and recital of a method which has been projected to obtain it with greater purity; a plan attended with the greateft national advantages, when you perufe the Bills of Mortality, and reflect how many die annually of putrid diforders. Among the lower claffes of people the ravages of this difeafe are peculiarly afflicting, as their poverty prevents them from obtaining bark, red wine, &c. which are ufually the medicines made ufe of. This important fubject has long been difcuffed through the medium of your excellent Journal; it has found in men of talents, patronage as well as perfecution;* the grand defideratum is yet undetermined, (and may remain fo a long time) whether it really poffeffes the powers afcribed to it. I truft, to the calm enquirer, fatisfactory anfwers may be found, both in my letters and in thofe of various others; for few, comparatively, have examined its merits; having, as they imagine, a fufficient remedy. Though thoufands die annually, they pafs unnoticed any new fuggeftion, and conceive, probably from the fimplicity of the fubject, it is altogether unworthy attention. As many have complained of the difagreeablenefs of taking yeaft in its original ftate, I was induced to confider of a method by which the quantity might be leffened without injuring its mode of action, and accordingly determined to filtrate a certain quantity, and take an exact account of what was loft by the procefs; accordingly, having obtained a quota of frefh fmall-beer yeaft, I proceeded as follows, viz.

A cup which I made ufe of, previous to being filled, weighed 2 oz. 6 dr. $\frac{1}{2}$; the cup full of yeaft weighed 8 oz. 5 dr. fo that the yeaft at firft, that is, before filtration, weighed 5 oz. 6 dr. $\frac{1}{2}$. I filtered it through common filtering paper, fuch as is tied round pill boxes, and let it remain twenty-four hours, at which time the beer had completely feparated, leaving a thick extract; and on weighing them, I found them as follows, viz.

Beer drawn away, weighed 1 oz. 1 dr.

Yeaft, 3 oz. $\frac{1}{2}$.

So I found I loft better than an ounce, probably by the paper having imbibed a large quantity, as well as allowing for fome that might not be taken off. This I do not confider a ftandard, as there may be much difference occafioned by the time the yeaft has remained after being taken from beer, and as it is occafionally adulterated by mixing beer with it, in order to increafe the value for fale. Having proceeded thus far, my next plan was to try whether the quantity being fmall or large

* See Mr. Cuftance's Letter, publifhed in No. XVII. for July, 1800.

large that was ſet apart, occaſioned any difference, and whether that quantity was beſt ſpread or otherwiſe. Into a four-ounce gallipot, I put on the 14th of October, 1800, two ounces of the pure extract, and ſet it in a dry ſituation; into a two-ounce gallipot I put half an ounce, and ſpread it round the ſides, ſo as to cover the whole of the inſide, thereby expoſing a much larger ſurface; and this I alſo laid in a very dry ſituation, namely, a cloſet by the fire: Into another two-ounce gallipot I put half an ounce, and laid it at the bottom in a lump; this, as well as the reſt, was carefully tied over with bladder and paper; but the latter I put in the dampeſt part of the cellar: Into a ſmall ſaucer I put half an ounce, and ſpread over the ſides; this was covered over and ſet near the fire, to bring it to a conſiſtence capable of pulverization. Thus far I proceeded on the 14th of October. On the 20th I examined the latter, and found it dried perfectly hard, of a nut brown colour, and ſtrong ſmell; and having procured half a pint of wort, juſt brewed, I heated it to the degree of 70, by the thermometer; and having previouſly mixed half a ſcruple of the powder with an ounce of the wort, and let them ſtand at a ſmall diſtance from the fire for an hour, I mixed them together: The half ounce of yeaſt that I ſet apart for drying, I omitted to mention, weighed before pulverization only twenty-three grains; thus loſing, by a ſecond evaporation, two hundred and ſeventeen grains from two hundred and forty.

On the next morning I examined the wort that had been mixed with the powder, but found the fermentation proceeded exceedingly ſlow, nor could I obtain from the proceſs any freſh yeaſt; I repeated my former experiment with the powder on October the 26th, but then found not the leaſt fermentation produced. Thinking another mode might be adopted that would better anſwer my expectations, I put a pint of water into a kettle, and having heated it to 75 of the thermometer, an ounce and a half of treacle was then put to it, which reduced it about five or ſix degrees; and having mixed a ſmall quantity of the liquor with the remaining powder, (about thirteen grains) I put them together, and after waiting ſome hours in anxious expectation, I confeſs I was much diſappointed by the reſult, when, on examination, I found it exactly as when mixed.

This experiment, however, has convinced me, that the gas is entirely diſſipated by being expoſed to heat; but a variety of reaſons induced me to try it: for, in the firſt place, the doſe of yeaſt in ſubſtance, and not freed from the beer, is about a large ſpoonful; and as the powder would have been given in much leſs compaſs, it might have been adminiſtered with greater pleasure to the patient, and certainty to the practitioner than in its original state; and secondly, had the fermenting properties of yeast been retained in the small compass of a powder, and possessing powers equally efficacious, the discovery would have been highly advantageous to the public; for yeast being sometimes at a most exorbitant price, and frequently not to be obtained for any, brewers as well as others, would have been enabled to keep by them a large stock, and the public obtain it at an easy rate. I have been very particular in informing you the exact method I adopted in placing it, both in damp and dry situations; for in one, I spread it round the inside of the pot, and in the other, I laid it in a lump. It would be trespassing on your time, to be scrupulously precise in enumerating the various processes that were afterwards made use of; but I am happy to say, I found the intention answer with little variation as to the place where they had been deposited, and I produced a copious fermentation from all the others at the different periods of a week, fortnight, and month, and till all was consumed. I think, from this narrative, it will plainly appear, that very great advantage is derived from filtration, as at least it enables the practitioner to prescribe with a greater degree of certainty, and the quantity lost by this plan, is known by referring to the former part of my letter. I was indebted for my knowledge of a press, to the ingenious Mr. Packer, of London, which is, a "*frame made very strong, and boards put inside, with grooves, and wound across the contrary way with string, in order to prevent a flannel bag, which is made the size of the press, and put inside it, filled with yeast, from getting into the grooves; by this means, an aperture is left for the beer to escape.*" According to Mr. P's account, about "*two-fifths beer is drawn away.*"

I have now closed the detail of my experiments with yeast. Some accounts of its unrivalled excellence in Typhus, have appeared in different Numbers of your instructive Journal, the authors of which, I believe, with the exception of one gentleman, have impartially confessed the beneficial effects. I have myself tried it in various instances, all of which recovered, though with appearances of rapid dissolution. The testimony of one might be considered as the effect, perhaps, of bigotry, or want of general information; but let the prejudiced mind peruse the ingenious letters of Dr. Lewin, of Liverpool, in No. IX. and Mr. Brown, of Hatton Garden, in No. XVI. The Doctor positively asserts, what I have so strenuously defended in opposition to the accusation of its enemies, "*that it does not affect the bowels;*" for I have tried it repeatedly, and always found it possessed of a contrary effect. Its use is not more more vehemently condemned even in this age of philofophy' than it was in March, 1683, when the Faculty of Medicine at Paris, folemnly maintained it noxious to the health of individuals; and though the bigotry of ignorant enthufiafts at that time was infufficient to abolifh its ufe, yet men of acknowledged abilities condemn, partly from intereft, partly from want of correct information, a medicine which they never made ufe of, or if they did, never attended to. When prejudice obfcures our refearches, and mankind fee through a falfe medium, can it be expected but condemnation muft enfue? It is fuch conduct that fetters inftruction, and prevents the foftering hand of difcovery from imparting its genial aid. As an auxiliary to the plan of treatment fpecified, I would recommend the ufe of the following for common drink, efpecially as large draughts of water are beneficial.

Take two glaffes of equal fize, or two pint pots, and having filled one with water, hold it over beer, while working and nearly fit to be tunned, and gently pour it from one glafs or pot into the other, for the fpace of an hour, and this will be found highly impregnated with fixed air; and as the latter poffeffes fuch a decided fuperiority in putrefactive tendency, this impregnated water muft, at leaft, be equally ferviceable.

It is obferved by Dr. Shebbeare, in his Theory and Practice of Phyfic, that fixed air has been very advantageoufly adminiftered againft putrid diforders in glyfters; and he alfo recommends the ufe of water impregnated with gas, as an antiputrefcent.

Thus far, Gentlemen, I have ftated the refult of my refearches: I have fcrutinized its various modes of action as circumfpectly as thofe who condemn, and what is here expreffed may be depended on. Convinced it will eventually remove the cloud of prejudice that has hitherto obfcured its glory, I leave the decifion to time and inveftigation, confcious that he who employs fome portion of his time in examining it with candour, will ultimately be repaid with ten-fold gratification.

I have the honour to be,

GENTLEMEN,

Your humble fervant,

*Winflow, Nov. 7, 1800.* I. H. GROSE.

P. S. I fhall take an opportunity, fome future day, to inform you of any additional difcovery.

